# Auranofin, a thioredoxin reductase inhibitor, causes platelet death through calcium overload

**DOI:** 10.1080/09537104.2017.1378809

**Published:** 2017-12-01

**Authors:** Matthew T. Harper

**Affiliations:** Department of Pharmacology, University of Cambridge, CB2 1PDCambridge, UK

**Keywords:** Necrosis, toxicology, redox, thrombosis, thrombocytopenia

## Abstract

Platelets are central to normal hemostasis and must be tightly controlled to prevent thrombosis. However, drug treatments that also affect platelets could lead to unwanted side effects on hemostasis or thrombosis. In this study, the effect of auranofin on platelets was tested. Auranofin, a gold-based thioredoxin reductase (TRXR) inhibitor, has been previously used in arthritis. Recently, auranofin and other inhibitors of the thioredoxin system have been proposed as novel anti-cancer therapies. TRXR is an important part of the antioxidant defenses in many cells that maintain intracellular proteins in their reduced state.

TRXR activity in platelets could be completely inhibited by auranofin. Auranofin-treated platelets showed several features of cell death, including the inability to aggregate in response to thrombin, leakage of cytosolic lactate dehydrogenase, and surface exposure of procoagulant phosphatidylserine. Auranofin increased platelet reactive oxygen species production and intracellular calcium concentration. DTT, a sulfydyl reducing agent, and BAPTA-AM, which chelates intracellular calcium, prevented auranofin-induced phosphatidylserine exposure. These data suggest that TRXR is an important part of the platelet antioxidant defense. TRXR inhibition by auranofin triggers oxidative stress and disrupts intracellular calcium homeostasis, leading to platelet necrosis. The use of auranofin or other TRXR inhibitors could therefore lead to unwanted side effects.

## Introduction

Platelets are central to normal hemostasis following vascular injury. Platelets rapidly adhere to sites of vascular damage and aggregate to form a multicellular plug [1]. A fraction of adherent, activated platelets undergo a necrosis-like cell death process triggered by intracellular calcium overload, mitochondrial dysfunction, and increased reactive oxygen species (ROS), resulting in surface exposure of phosphatidylserine (PS) [2–5]. Platelet PS forms a procoagulant surface that accelerates thrombin generation and blood coagulation [6]. Whilst platelet procoagulant activity is important for normal hemostasis, it is also involved in the pathogenesis of arterial thrombosis on ruptured atherosclerotic plaques [7]. For this reason, platelet intracellular calcium concentration and ROS must be tightly regulated to prevent inappropriate coagulation.

Cells are protected from ROS by multiple antioxidant defense systems, which maintain proteins inside of the cell in a reduced state with many free sulfhydrol groups and few disulfide bonds [8]. The major antioxidant disulfide reductases that maintain proteins in their reduced state are the thioredoxin reductase/thioredoxin system and the glutathione reductase/glutathione system [9,10]. Thioredoxin (TRX) is a dithiol-disulfide reductase that catalyzes the reduction of disulfide bonds in its substrates. The active site disulfide in TRX is maintained in its reduced state by thioredoxin reductase (TRXR) in a NADPH-dependent manner [9,11]. Thus, the intracellular reducing function of TRX is dependent on the activity of TRXR [10]. In addition to maintaining intracellular proteins in their reduced state, TRX protects cells from oxidative stress by acting as an electron donor for thioredoxin peroxidases that catalyze the reduction of hydrogen peroxide [10].

The TRXR–TRX system is an attractive clinical target. Auranofin, a gold-based TRXR inhibitor, has been used to treat arthritis, but recently it has been proposed that auranofin or other TRXR–TRX system inhibitors could be potential anti-cancer agents [12–15]. For example, auranofin triggers caspase-dependent apoptosis in promyelocytic leukemia cells [16,17] and insulinoma cells [18]. However, the clinical use of such an approach will depend on how other cells of the body are affected. In this study, the role of the TRXR–TRX system in protecting platelets from oxidative stress was investigated. Platelets were found to express constitutively active TRXR. Inhibition of TRXR induced increased platelet ROS, disrupted intracellular calcium homeostasis and led to platelet necrosis. This could lead to unwanted side effects during use of TRXR–TRX-targeted therapy.

## Methods

### Materials

Auranofin, A23187 and BAPTA-AM were from Tocris (R & D Systems, Abingdon, UK), Q-VD-OPh (non-O-methylated) was from Calbiochem (Merck Millipore, Nottingham, UK). CM-H_2_DCFCA and Nu-PAGE sample buffer were from Invitrogen (Life Technologies, Paisley, UK). Anti-caspase-3 and anti-gelsolin antibodies were from Cell Signaling Technology (Beverley, MA, USA). Annexin V-FITC was from Abcam (Cambridge, UK). Fluo-2 LR-AM (acetoxymethylester) was from Teflabs (Cambridge Bioscience, Cambridge, UK). The lactate dehydrogenase (LDH) assay kit was from BioVision inc. (Milpitas, CA, USA). All other reagents, including the TRXR assay kit (CS0170), were from Sigma (Poole, UK), and were of analytical grade.

### Platelet preparation

Blood was obtained from healthy drug-free volunteers with approval from the local Research Ethics Committee of the University of Bristol, UK; informed, written consent was obtained in accordance with the Declaration of Helsinki. All donors reported that they not taken any drugs known to affect platelet function (for example, aspirin) for 10 days prior to donation.

Platelet rich plasma was prepared by centrifugation (180*g*, 17 min). Platelets were pelleted by centrifugation (550 g, 10 min) in the presence of PGE_1_ (140 nM) and resuspended in HEPES-buffered saline (HBS) (135 mM NaCl, 3 mM KCl, 12 mM 10 mM Hepes, 5 mM glucose, 1 mM MgCl_2_, and 0.02 U.ml apyrase [grade VII], pH 7.3) to a density of 4 × 10^8^/ml. Unless indicated otherwise, all experiments were performed at 37 °C in the presence of 1 mM extracellular CaCl_2_.

### Thioredoxin reductase (TRXR) activity assay

Platelets (4×10^8^/ml) were treated with auranofin at the concentrations indicated for 30 min then lysed on ice for 30 min by addition of an equal volume of Triton X-100 (2 % v/v in HBS). TRXR activity was monitored using a commercial assay kit according to the manufacturer’s instructions. In this assay, TRXR activity is monitored as the ability to reduce 5,5ʹ-dithiobis(2-nitrobenzoic) acid with NAPDH to 5-thio-2-nitrobenzoic acid, which was measured by absorbance at 412 nm. Samples were analyzed in the presence and absence of an inhibitor of TRXR, to determine the reduction due only to TRXR activity.

### Platelet aggregation

Aggregation of thrombin-stimulated platelets were monitored in an optical aggregometer (Chrono-Log, Labmedics, Manchester, UK) at 37°C, with continuous stirring at 1000 rpm.

### Lactate dehydrogenase (LDH) release

Platelets (2×10^8^/ml) were stimulated as indicated and then pelleted by centrifugation (13,000*g*, 2 min). LDH activity in the supernatant was detected using a commercially available assay kit according to the manufacturer’s instructions.

#### Annexin V binding

**Figure 1.**
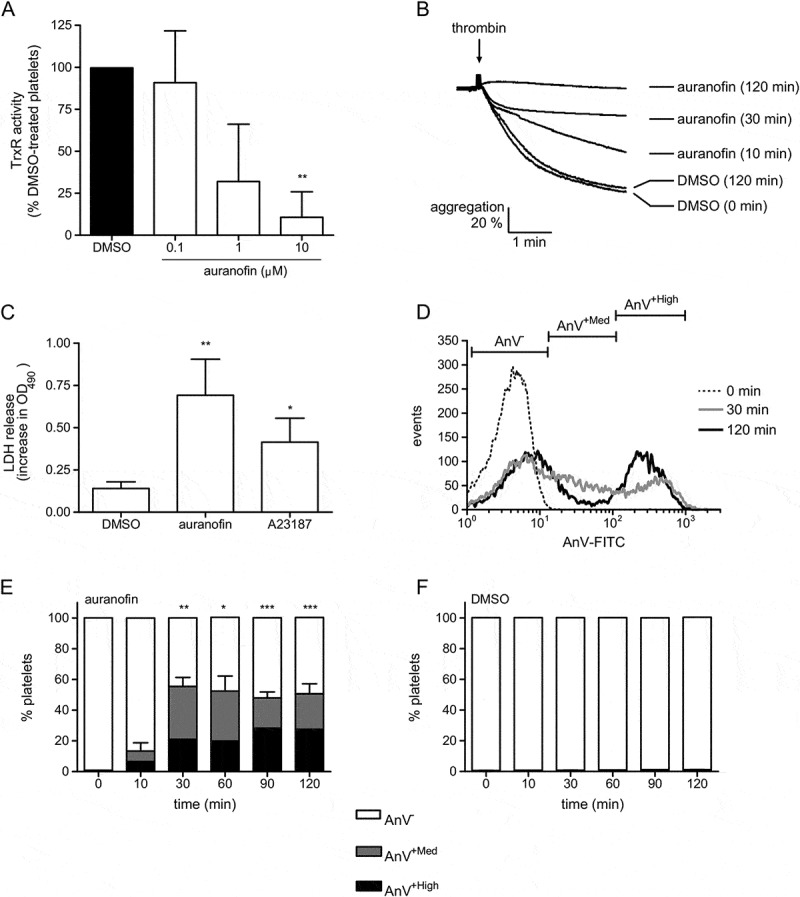
Auranofin induces death of platelets. (A) Washed human platelets were treated with the indicated concentration of auranofin or the vehicle (DMSO) for 30 min prior to lysis. TRXR activity in platelet lysates is shown (*n* = 4; ** *p* < 0.01 compared to DMSO-treated control). (B) Platelets were treated with auranofin (10 µM) or DMSO under non-stirring conditions. After the indicated time, platelets were stimulated with thrombin (1 U/ml) with stirring. The traces are representative of 3 independent experiments. (C) Platelets were treated with auranofin, the Ca^2+^ ionophore A23187 (10 µM) or DMSO for 2 hours. LDH activity released into the medium was determined and is expressed as the increase in optical density at 490 nm (OD_490_) (*n* = 3; * *p* < 0.05, ** *p* < 0.01 compared to DMSO-treated control). (D) Representative histogram of platelets treated for the indicated times with auranofin and stained with annexin V (AnV). The gating of each population is indicated. The mean percentage of each of population at various times is shown in (E) (*n* = 5; * *p* < 0.05, ** *p* < 0.01, *** *p* < 0.001 for AnV^+High^ at indicated time compared to 0 min). For comparison, AnV staining of control platelets treated with DMSO is shown in (F) (*n* = 3).

Annexin V (AnV)-FITC was used to detect surface PS exposure. Platelets (1 × 10^8^/ml) were stimulated in the presence of CaCl_2_ (1 mM) for the indicated times. 10 µl platelets were then added to 40 µl HBS containing 2.4 mM CaCl_2_ and 1 µl AnV-FITC for 2 min, then fixed with 1 % paraformaldehyde on ice. Using this approach, no increase in AnV binding was seen in unstimulated samples (data not shown, but see Figure 1F). Fluorescence was detected by flow cytometry, with platelets gated by their forward and side scatter profile. Analysis of 20,000 platelets was performed using a Becton Dickinson LSRII. Data were analyzed using Flowing Software 2.4 (Turku Centre for Biotechnology, Finland).

### Immunoblotting

Platelet samples (4 × 10^8^/ml) were solubilized in Nu-PAGE sample buffer. Proteins were resolved by electrophoresis using 12% SDS-PAGE. Samples were then transferred to PVDF membrane, blocked with 5 % bovine serum albumin, and subjected to immunoblotting with anti-gelsolin or anti-caspase-3 antibodies.

#### Reactive oxygen species (ROS) and intracellular calcium concentration measurements

Washed platelets (1×10^8^/ml) were incubated with the ROS-sensitive fluorescent dye, CM-H_2_DCFCA (10 µM, 30 min, 37 °C), followed by treatment with the indicated inhibitors for 20 min. For intracellular Ca^2+^ measurements, PRP was incubated with the Ca^2+^-sensitive fluorescent dye, Fluo-2 LR-AM (10 µM, 30 min, 37 °C), prior to washed platelet preparation as above. Platelets were stimulated with auranofin in a Fluoroskan Ascent microplate fluorometer (Thermo Scientific, Loughborough, UK). Fluorescence was excited at 488 nm and monitored at 520 nm, and normalized to the initial fluorescence (F/F_0_).

### Data analyses

Mean data are presented with standard errors; the number of replicates of each experiment, representing independent experiments from platelet preparations from different donors is reported in the figure legends. Data were analyzed by one-way ANOVA, with Dunnett’s test for multiple comparisons, or by two-way ANOVA with Bonferroni post-test, as appropriate, using Prism 4.0 software (Graphpad Inc. La Jolla, CA, USA). *p* < 0.05 was considered statistically significant.

## Results

### Auranofin, a TRXR inhibitor, induces platelet necrosis

TRXR activity was detected in lysates of unstimulated human platelets (Figure 1A). To assess the role of TRXR, the TRXR inhibitor, auranofin, was used. Auranofin is a gold-based drug that has been used to treat rheumatoid arthritis and is able to block TRXR activity in many cells. This was also found to be the case in platelets. Auranofin treatment for 30 minutes resulted in a concentration-dependent reduction in TRXR activity. 10 µM auranofin was sufficient to completely block TRXR activity (Figure 1A; *n* = 3).

Auranofin has been reported to induce death of many different cell types. Platelet dysfunction and death was assessed by several approaches. First, the effect of auranofin on the capacity of platelets to respond to stimulation was determined. Platelets were treated with auranofin (10 µM) for up to 2 hours (or DMSO as vehicle control) then stimulated with thrombin (1 U/ml). DMSO-treated platelets underwent rapid and extensive aggregation, as indicated by the decrease in optical density (Figure 1B). In contrast, platelets treated with auranofin for 2 hours did not aggregate. Second, lactate dehydrogenase (LDH) release was determined as a marker of loss of plasma membrane integrity. LDH was detected in the medium following auranofin treatment, whereas treatment with DMSO had no significant effect (Figure 1C). LDH release was also in platelets treated with the Ca^2+^ ionophore, A23187 (10 µM). Ca^2+^ ionophores have been previously shown to induce platelet necrosis [19]. Third, treatment with auranofin also resulted in a time-dependent increase in annexin V (AnV) binding, indicative of PS exposure and platelet procoagulant activity (Figure 1D–E). The AnV-positive fraction (AnV^+Total^) could be resolved into two populations, designated AnV^+Med^ and AnV^+High^. PS exposure was not due to prolonged platelet incubation at 37 °C, since treatment with DMSO did not induce AnV binding even up to 2 hours (Figure 1F).

### Auranofin-induced platelet death is independent of caspases

**Figure 2.**
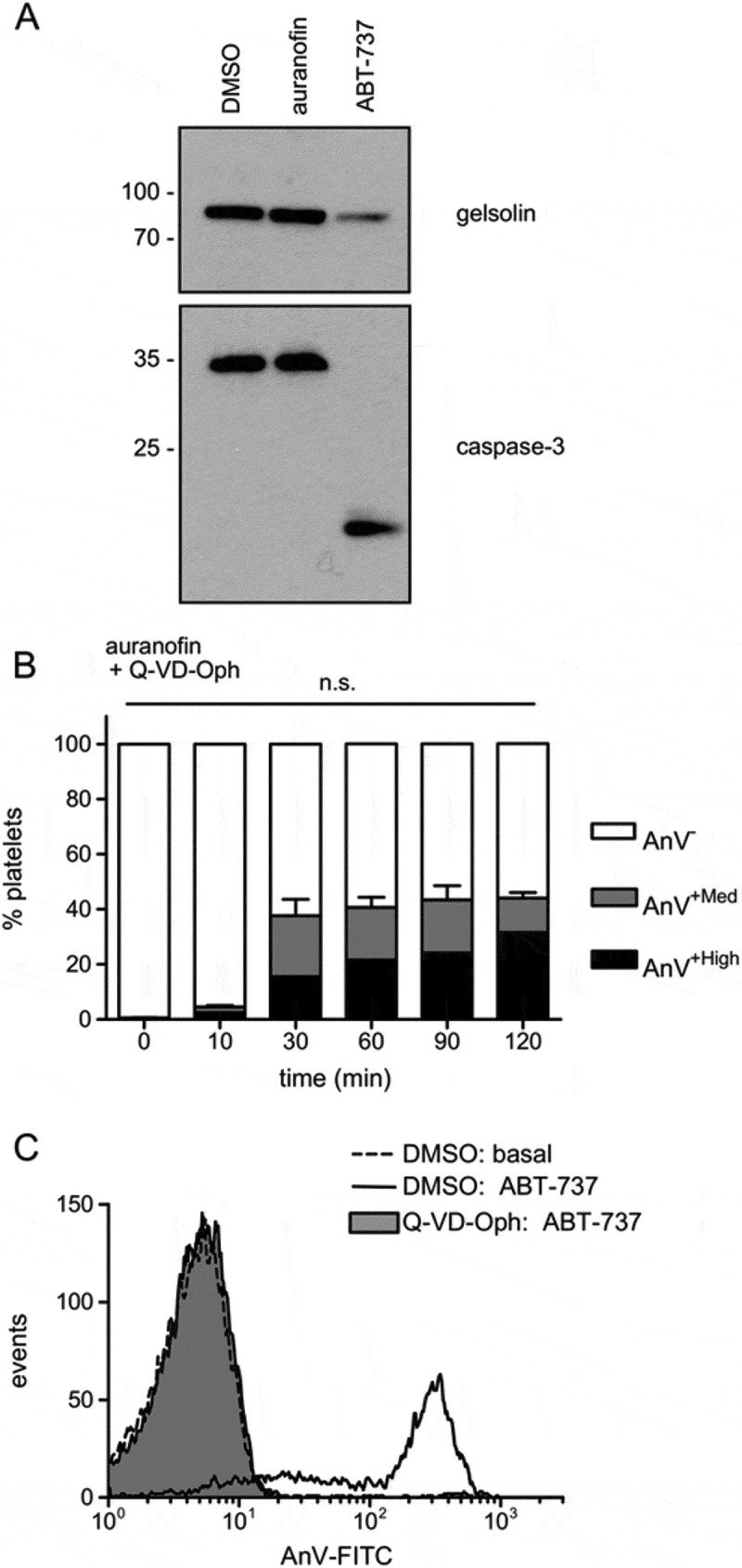
Auranofin-induced PS exposure is caspase-independent. (A) Platelets were treated with auranofin, ABT-737 (10 µM) or DMSO as control for 2 hours. Gelsolin and caspase-3 cleavage was detected by immunoblotting. Approximate positions of molecular weight markers are shown on the left. The blot is representative of three independent experiments. (B) Platelets were treated with the pan-caspase inhibitor, Q-VD-Oph (50 µM) prior to treatment with auranofin. Mean percentage of AnV^+High^ and AnV^+Med^ populations is shown (*n* = 4; n.s. not significantly different to auranofin-treated platelets with DMSO instead of Q-VD-Oph). (C) Representative histogram of platelets treated with Q-VD-Oph or DMSO, then with ABT-737 for 2 hours (*n* = 4).

Caspase-dependent apoptosis can lead to PS exposure in platelets (Schoenwaelder SM et al, 2009; Vogler M et al, 2011). However, auranofin did not induce caspase-3 activation in platelets, as assessed by cleavage of caspase-3 or its substrate, gelsolin (Figure 2A). In contrast, the apoptosis-inducing BH3 mimetic, ABT-737 [20], did trigger cleavage of caspase-3 and gelsolin. Consistent with these observations, the pan-caspase inhibitor, Q-VD-Oph, had no effect on auranofin-induced AnV binding (Figure 2B), whereas it did prevent ABT-737-induced AnV binding (Figure 2C). These data indicate that auranofin-induced platelet death was not a result of caspase-dependent apoptosis.

### Auranofin increases ROS in unstimulated platelets

**Figure 3.**
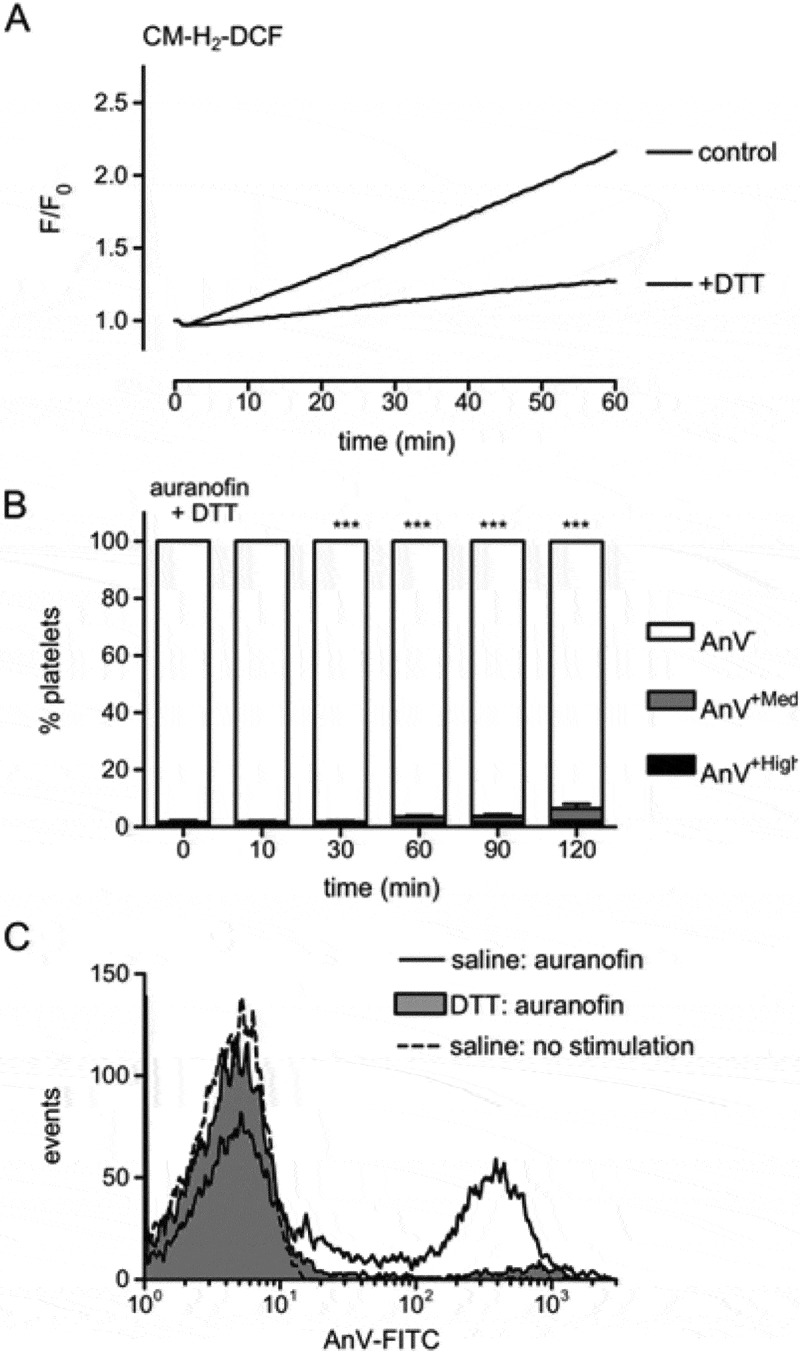
Auranofin increase ROS. (A) CM-H_2_DCFDA-loaded platelets were treated with the sulfhydryl reagent, DTT (1 mM), or the vehicle (saline) prior to addition of auranofin. The trace is representative of 4 independent experiments. (B) Platelets were treated DTT, prior to treatment with auranofin for the indicating times, and then stained with AnV. Mean percentage of AnV^+High^ and AnV^+Med^ populations is shown (*n* = 4; *** *p* < 0.001 compared to auranofin-treated platelets with saline instead of DTT). A representative histogram of platelets treated with auranofin for 2 hours is shown in (C).

Since a major role of the TRXR/TRX system is to protect cells from protein sulfhydyl oxidation, the effect of DTT (1mM), a sulfhydyl reducing agent, on auranofin-induced AnV binding was tested. DTT completely abolished both AnV-binding populations (Figure 3B–C), indicating that sulfhydyl oxidation is involved in the effects of auranofin. In addition, the effect of auranofin on platelet ROS accumulation was tested, since the TRXR/TRX system protects some cells from ROS . In CM-H_2_DCFDA-loaded platelets, auranofin treatment induced a rapid increase in fluorescence, indicating an increase in hydrogen peroxide (Figure 3A). This was also significantly inhibited by DTT, suggesting that protein sulfhydyl oxidation leads to ROS accumulation.

### Auranofin disrupts intracellular calcium homeostasis

**Figure 4.**
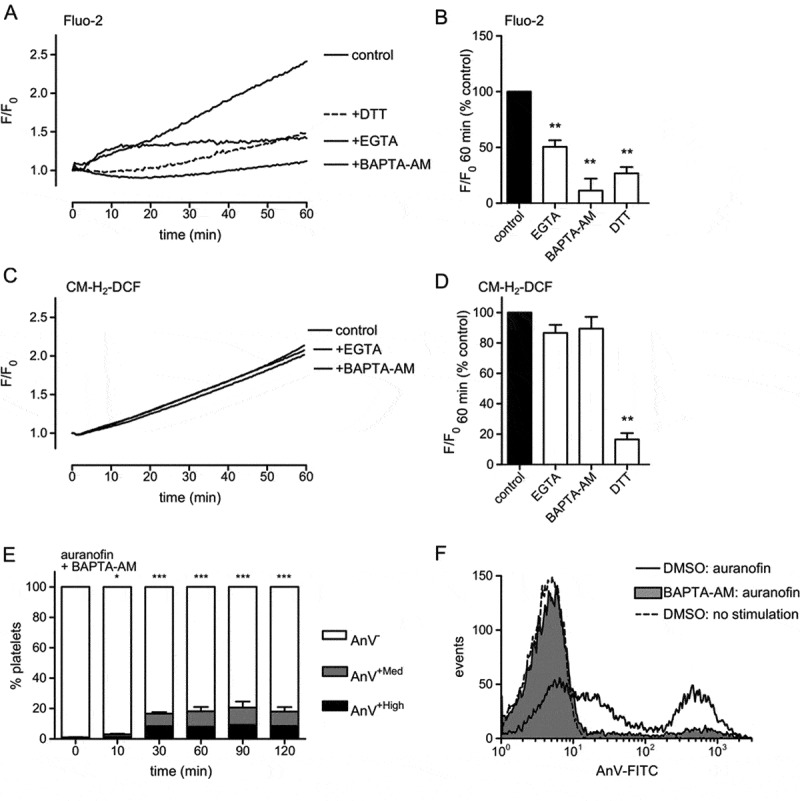
Auranofin induces PS exposure by increasing intracellular calcium. Fluo-2 LR-loaded platelets (A–B) or CM-H_2_DCFDA-loaded platelets (C–D) were treated with DTT (1 mM) or BAPTA-AM (20 µM) for 20 min prior to addition of auranofin. Some samples had extracellular EGTA (1 mM) in place of CaCl_2_. Representative traces are shown in (A, C), and mean data (+ S.E.) are shown in (B, D) (*n* = 4; ** *p* < 0.01 compared to vehicle-treated control). (E) Platelets were treated BAPTA-AM, prior to treatment with auranofin for the indicating times, and then stained with AnV. Mean percentage of AnV^+High^ and AnV^+Med^ populations is shown (*n* = 4; * *p* < 0.05, *** *p* 0.001 compared to auranofin-treated platelets with DMSO instead of BAPTA-AM). A representative histogram of platelets treated with auranofin for 2 hours is shown in (F).

A large or sustained increase in intracellular Ca^2+^ concentration can trigger necrosis-like platelet death [4]. To investigate the possible role of intracellular calcium in the effects of auranofin, platelets were loaded with the Ca^2+^-sensitive due, Fluo-2LR. Auranofin induced a sustained increase in intracellular Ca^2+^ concentration (Figure 4A). This was completely prevented by pre-treating platelets with BAPTA-AM (Figure 4A–B). In the absence of extracellular Ca^2+^ (and with EGTA added), the Ca^2+^ signal was substantially reduced but not completely abolished, indicating that auranofin induces both Ca^2+^ release from intracellular stores and Ca^2+^ entry. The increase in intracellular Ca^2+^ concentration was also completely blocked by DTT, indicating that protein sulfhydyl oxidation is likely to be the cause. In contrast, neither extracellular EGTA nor BAPTA-AM treatment had a significant effect on platelet ROS accumulation (Figure 4C–D). In BAPTA-loaded platelets, AnV^+Total^ was significantly inhibited, though not completely (Figure 4E–F).Together, these data suggest that auranofin treatment results in oxidation of protein sulhydryl groups leading to intracellular Ca^2+^ release and Ca^2+^ entry, which triggers Ca^2+^-dependent PS exposure and platelet necrosis.

## Discussion

**Figure 5.**
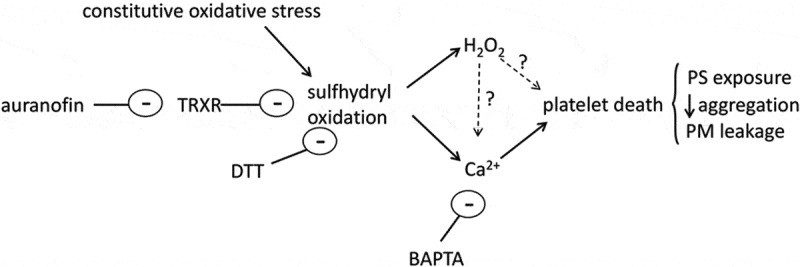
A hypothesis for the action of auranofin on platelets. The thioredoxin reductase (TRXR) maintains the reduced state of intracellular proteins in the face of constitutive oxidative stress. Auranofin inhibits TRXR, leading to oxidation of protein sulfhydrol groups. This leads to an increase in intracellular Ca^2+^ concentration, through Ca^2+^ release and Ca^2+^ entry, and accumulation of hydrogen peroxide (H_2_O_2_). The increased intracellular Ca^2+^ concentration leads to platelet death. Whether the H_2_O_2_ also contributes to platelet death is unclear.

Platelets that are exposed to cytosolic Ca^2+^ overload or excessive ROS accumulation can undergo necrotic cell death [4]. During thrombosis, this process is triggered by signals of vascular damage, such as collagen and thrombin, and the resulting exposure of procoagulant PS on the platelet surface accelerates further thrombin generation and coagulation [1]. This process is important both in normal hemostasis and in pathological arterial thrombosis. Conversely, it is important for platelets that platelets do not undergo necrotic death in the absence of vascular damage. One novel finding of this study is that apparently unstimulated platelets must be continuously protected by the TRXR–TRX system. Without this protection, intracellular Ca^2+^ concentration increases, triggering necrotic platelet death. This is summarized in Figure 5.

Platelet death is often described as apoptosis, but not all occurrences of platelet death warrant this term (Kile, 2014). Auranofin-induced platelet death appears to be independent of caspases, since activation of the executioner caspase-3 could not be detected and PS exposure was not affected by caspase inhibition. This suggests that although platelets contain the machinery of intrinsic apoptosis [21], which can be triggered by the BH3 mimetic ABT-737 (Figure 2 and [20,22]), auranofin-induced platelet death is not through apoptosis. In contrast, auranofin results in caspase-dependent apoptosis in promyelocytic leukemia cells [16,17] and insulinoma cells [18], suggesting that the manner of cell death may dependent on the cellular context.

The protection afforded to platelets by the TRXR–TRX system can be mimicked by the sulfhydryl reagent, DTT, suggesting that a major function of TRXR–TRX in this system is to maintain the reduced state of intracellular proteins. This is a major function of the TRXR–TRX system in many other cell types [9,10]. TRX may also regulate platelet activation by controlling the redox state of GPIb and GPVI [23]. Acute (10 minutes) inhibition of TRX reduced platelet aggregation in response to cross-linked collagen-related peptide [23]. Although this could point to toward repurposing TRX inhibitors as anti-thrombotics, the necrotic effect of longer disruption of the TRXR/TRX system (2 hours in this study) indicates that there may be unwanted effects to this approach.

Auranofin treatment disrupts intracellular Ca^2+^ homeostasis and increases intracellular hydrogen peroxide. Whether these two events are mechanistically linked is not clear. Similar increases in intracellular Ca^2+^ concentration have been observed in platelets treated with exogenous hydrogen peroxide [24,25], suggesting that ROS could contribute to the disrupted Ca^2+^ homeostasis. The observation that EGTA does not completely prevent the increase in intracellular Ca^2+^ concentration suggests that Ca^2+^ is released from intracellular Ca^2+^ stores. The increase is intracellular Ca^2+^ concentration is amplified by Ca^2+^ entry. This may represent store-operated Ca^2+^ entry subsequent to depletion of the intracellular Ca^2+^ stores [26], although it is possible that ROS may directly activate Ca^2+^ entry [25,27].

By increasing intracellular Ca^2+^, auranofin induced PS exposure in a substantial fraction of platelets. Platelet PS exposure is important in thrombosis because it forms a procoagulant surface that accelerates vascular thrombin generation and coagulation. One consequence of auranofin treatment could be to induce a prothrombotic state. However, another feature of platelet necrosis was the inhibition of platelet aggregation (Figure 1), which would tend to reduce the prothrombotic effect and could instead reduce hemostasis. Moreover, thrombocytopenia is an uncommon, but potentially severe, known side effect of auranofin treatment [28,29]. This has also been observed with a novel TRXR-1 inhibitor, PX-916, which was reported show strong anti-tumor activity in a number of animal models but with thrombocytopenia – an approximately 45% reduction in platelet count – noted as a major toxicity [30]. Thrombocytopenia would also reduce hemostasis. A similar effect is seen with the BH3 mimetics, ABT-737 and ABT-263, which induce caspase-dependent apoptosis, resulting in PS exposure but also reduced platelet adhesive function and thrombocytopenia in mice. The net result of BH3 mimetics is that arterial thrombosis is reduced and bleeding time is increased [31]. Although auranofin triggers platelet death through a different mechanism to these BH3 mimetics, there is a similar loss of platelet aggregation responses, suggesting that there may be a similar disruptive effect on hemostasis.

The pathogenesis of auranofin-induced thrombocytopenia is not clear [28]. Although an increase in anti-platelet antibodies and immune-mediated platelet destruction is a likely factor in the thrombocytopenia induced by gold compounds [29,32,33], an increase in platelet necrosis may also contribute. Surface-exposed PS acts as an ‘eat-me’ signal to promote clearance of dead or dying cells by macrophages and other scavenger cells [34]. Increased platelet PS could therefore promote platelet clearance and thrombocytopenia. Interestingly, increased oxidative stress has also been associated with the development of immune thrombocytopenia, since oxidized proteins can induce autoantibody production [35]. Conversely, increased markers of platelet death including PS exposure have been reported in pediatric immune thrombocytopenia [36]. It is possible, therefore, that necrotic platelet death involving oxidative stress and immune-mediated thrombocytopenias may be linked in a complex manner.

In summary, this study shows that inhibition of TRXR in unstimulated platelets by auranofin can induce oxidative stress, disruption of intracellular Ca^2+^ homeostasis, and leads to necrotic platelet death. There is increasing interest in the development of inhibitors of the TRXR–TRX system in new clinical situations, for example as novel anti-cancer agents. However, the important role of TRXR in protecting platelets means that disruption of this system could lead to unwanted side effects in platelet count or function.
